# The Wiring Economy Principle: Connectivity Determines Anatomy in the Human Brain

**DOI:** 10.1371/journal.pone.0014832

**Published:** 2011-09-07

**Authors:** Ashish Raj, Yu-hsien Chen

**Affiliations:** 1 Department of Radiology, Weill Cornell Medical College, New York, New York, United States of America; 2 Department of Computer Science, Cornell University, Ithaca, New York, United States of America; Cajal Institute, Consejo Superior de Investigaciones Científicas, Spain

## Abstract

Minimization of the wiring cost of white matter fibers in the human brain appears to be an organizational principle. We investigate this aspect in the human brain using whole brain connectivity networks extracted from high resolution diffusion MRI data of 14 normal volunteers. We specifically address the question of whether brain anatomy determines its connectivity or *vice versa*. Unlike previous studies we use weighted networks, where connections between cortical nodes are real-valued rather than binary off-on connections. In one set of analyses we found that the connectivity structure of the brain has near optimal wiring cost compared to random networks with the same number of edges, degree distribution and edge weight distribution. A specifically designed minimization routine could not find cheaper wiring without significantly degrading network performance. In another set of analyses we kept the observed brain network topology and connectivity but allowed nodes to freely move on a 3D manifold topologically identical to the brain. An efficient minimization routine was written to find the lowest wiring cost configuration. We found that beginning from any random configuration, the nodes invariably arrange themselves in a configuration with a striking resemblance to the brain. This confirms the widely held but poorly tested claim that wiring economy is a driving principle of the brain. Intriguingly, our results also suggest that the brain mainly optimizes for the most desirable network connectivity, and the observed brain anatomy is merely a result of this optimization.

## Introduction

The human brain is believed to have faced evolutionary pressure to optimize various network quantities, for instance information capacity, latency, average shortest path length [1], clustering tendencies [2], complexity [3] and wiring cost. The latter property is especially important because it determines the total metabolic cost associated with maintaining such a large scale network. The economy of wiring in physical systems has been analyzed from a network viewpoint [4], [5]. The metabolic costs of building and functionally resourcing the brain are large in proportion to the total energy budget of the body [5]. Van Essen and Stevens [6] first conjectured that axonal tension might cause strongly connected regions to pull towards each other during development, thereby leading to compact neural circuitry and short wiring length. Computational modeling studies [4] showed that graphs computationally evolved for high complexity of dynamic behavior had a sparse, small-world topology of edges between connected nodes. Network analysis of macaque brain data [7] appear to support wiring economy principle, albeit partially.

Several studies at the level of small and specific neuronal populations have reported preference for low wiring cost in the brain [8], [9]. Most notably, Chklovskii et al. [10]–[12] demonstrated that the optimal component placement which minimizes total wiring cost agrees with results from specific regions of the neocortex. It is further hypothesized that the diversity of interneurons in the mammalian cortex reflects a compromise between computational needs and wiring economy [10], [13]. However, few studies on this topic have used human brain connectivity data.

Although a large number of studies and review papers have appeared that all speculate that wiring cost minimization must be a driving principle behind the observed network topology (meaning how the nodes are connected to each other) - whether small world [14], heavy-tailed degree distributions [15] or hierarchical, see [1], [15] for example - these assertions have yet to be objectively verified. Some authors have found brain networks from functional MRI studies to have high efficiency at low cost [15], where cost was defined as the number of connections, after binarization, of the network, assuming this to be directly related to the metabolic cost of maintaining such a network. However, this can only be an approximation, since the actual cost of a network must depend on the number, strength and total length of connections.

In this paper we report new results on wiring economy of whole human brain networks and shed new light on the relationship between brain anatomy and connectivity. We extract weighted networks from human diffusion MRI (dMRI) data by modifying the method of [16] with a robust statistical thresholding technique to remove statistically questionable connections. We propose a novel wiring cost metric and show that the human brain network significantly minimizes it compared to any equivalent random network. During this analysis the locations of the nodes is kept constant. In another set of analyses we consider it a given that the brain has optimized for the most desirable topological properties, which we hold constant and allow node placement to vary. We address the question: does the minimum wiring placement of nodes have any resemblance to the brain? We found, surprisingly, that the answer is affirmative. To our knowledge these results have not been reported before, and provide the most complete empirical support for the wiring efficiency conjecture. We also show support for the intriguing notion that the observed cortical anatomy uniquely results from the requirement of delivering the observed network topology at the cheapest wiring cost. The suggestion that network connectivity is of primary importance while the anatomy is merely a result of connectivity, sheds new light on the structure-function relationship of the brain.

### Note on dMRI-based networks

Due to its relative novelty, dMRI-based networks have not so far been well-analyzed for wiring economy, with a couple exceptions - see Discussion. New developments in dMRI Tractography make it possible to infer whole brain structural connections. There are many advantages to dMRI-derived networks in investigating wiring cost. In contrast to tracer-based studies these networks are weighted (have real-valued weights) and are available for human subjects. Tracer-based connections are typically reported as binary off-on connections, or assume one of 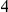 possible ordinal values from 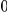 to 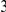. Although they are somewhat coarse, with only about 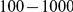 nodes representing large cortical regions, they provide estimate of connection strength between cortical regions given by the number or density of large fiber bundles. Since these techniques are non-invasive they provide the only practical means of interrogating structural human brain networks *in vivo*. Brain networks extracted from dMRI data were shown to have cost-efficient connections giving so-called small world networks, a term denoting a specific clustering and path length attribute of a network. Their network weights were also found to have “heavy-tailed” distributions [16], [17].

### Notations

We define a network 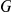 with 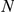 nodes as a set of nodes 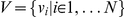 and a set of edges given by an ordered node pair 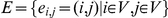. Each edge has a connection weight given by 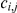. We define the *connectivity strength* of a node 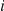 in this graph as the sum of all connection weights termkinating at 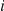:
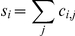
In a brain network each node has a location on either the neocortex or for deep brain gray matter in the interior, referred to as 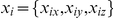 the 3D coordinates of node 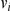. Note that dMRI does not allow us to measure the directionality of brain networks, even though individual neurons are known to be directional. It is generally the case that major fiber bundles resolvable by dMRI, especially cortico-cortical pathways, have roughly equal number of connections in either direction [18].

In this paper we use the shorthand *connectivity* to mean the set of edge weights 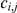 of the network, and *topology* as shorthand for the set of (unweighted) edges 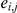. We will denote matrices by capital letters and vectors by **boldface** letters.

## Results

The average connectivity matrix from 14 subjects after significance thresholding at level 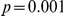 is in Figure S1. It has 233 non-zero entries out of possible 
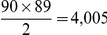
. The actual connectivity data of all 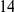 subjects are being included as the binary file “connectivity-matrices.mat” in Supporting Information S2.

### Wiring cost I: Constant node placement, varying connectivity

**Figure 1:**
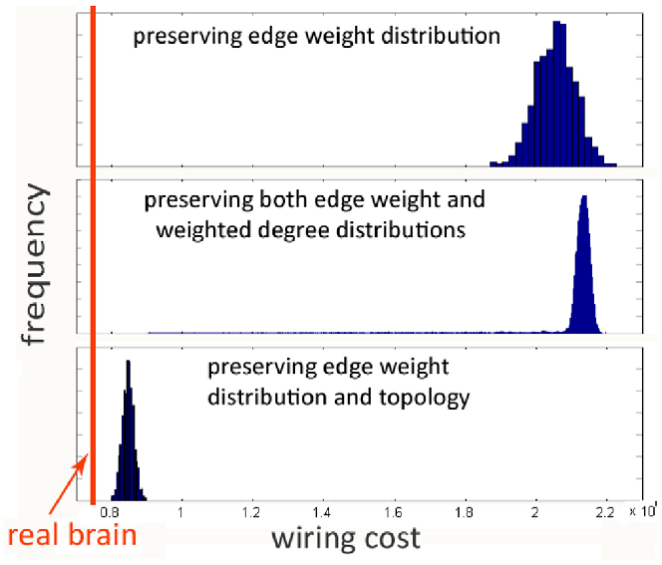
Histogram of wiring cost. Top: random networks with the same edge weight distribution as the brain. Middle: random networks with the same weight distribution as well as weighted node degree distribution as the brain. Bottom: random networks with the same weight distribution as well as topology as the brain. Wiring cost of the real brain network is shown by the red vertical bar for comparison.

**Figure 2:**
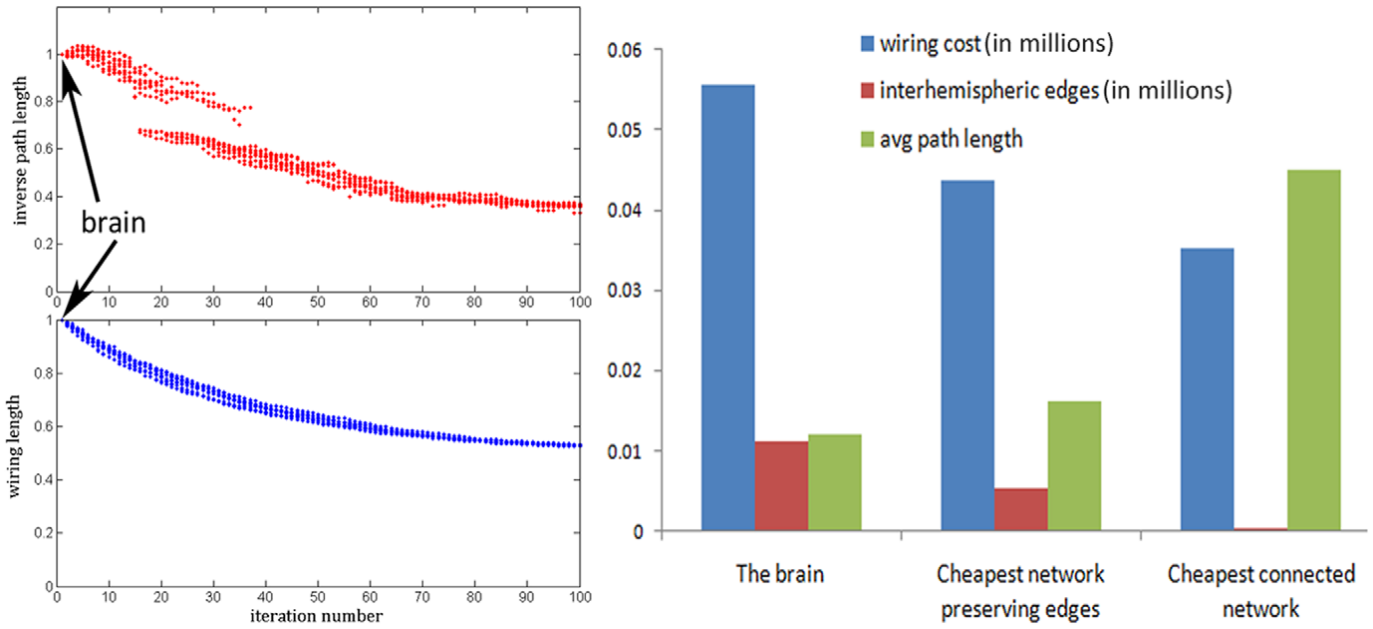
Cheaper-than-brain networks. *L*eft: Normalized wiring cost and path length of cheaper-than-brain networks, obtained from 10 runs of Algorithm A4. Cheaper wiring comes with reduced path length. The jump in path length is a result of the network splitting into two pieces. *R*ight: Wiring cost, total inter-hemispheric connection weight and average path length of the brain and two contrived networks with cheaper wiring cost. Cheaper wiring is achieved by redirecting inter-hemispheric connections to sub-cortical ones, which results in higher path length. The wiring cost and connection weights are in units of millions.

Figure 1 shows histograms of the wiring cost of 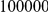 random networks, in comparison to the brain wiring cost. Part (a) shows results for random networks with preserved weight histogram, and part (b) for those which preserve both weight histogram as well as degree distribution. The brain's wiring cost is much smaller than almost any random network of either type. However, it is possible to obtain cheaper wiring cost than the brain, using algorithms specifically designed to do so. In Figure 2 we show that wiring cost of such networks, obtained from two such algorithms (see Methods), can be lower than the brain's. However, cheaper wiring comes at the cost of highly reduced network performance, as measured by average path length, which is much greater for these contrived networks than for the brain. We also observe that this reduction in wiring cost was specifically achieved by redirecting most of the inter-hemispheric connections to sub-cortical connections.

### Wiring cost II: Constant connectivity, varying placement

**Figure 3:**
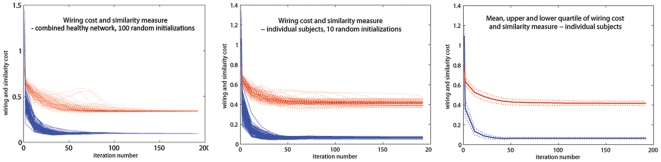
Convergence performance of wiring cost minimization algorithm. Part (a) shows cost function of combined healthy network after 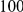 random starting configurations, (b) shows cost over individual subjects' networks, after 10 random initializations, and (c) shows the mean (bold curve), upper and lower quartiles (dotted lines) of (b). Both the wiring cost (in blue, (2)) and a measure of similarity to the brain anatomic configuration (in red) are shown. The y-axis is in arbitrary units, after normalizing each quantity by the value at the initial random configuration. Note that although the algorithm only minimizes for wiring cost, the similarity measure is also getting optimized.

Figure 3 depicts 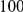 randomly initialized runs of the cost function (2) being minimized by Algorithm A6. As the wiring cost metric is successively reduced, the network embedding is getting closer and closer to the true brain configuration as measured by the brain similarity index, calculated as follows (lower value means more similar to the brain). The similarity index has to be rotation-invariant and insensitive to actual node locations, since our formulation does not require that node locations coincide with the actual brain. Therefore we developed an index based on the “local neighborhood” of each node: We ask how many nearest 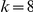 neighbors seen by each node agree between the computed cortical configuration and the true brain configuration? This allows us to compare the similarity between the two configurations without using a Euclidean distance. We show results for the combined healthy network as well as individual subjects' networks (in Supporting Information S1), after several random initializations. Observe that regardless of initialization, the algorithm converges to the same cost function, and also converges to the same similarity measure. This implies that a strong local minimum, or even the global minimum has been found. The mean, upper and lower quartiles are also indicated, and suggest that the process is consistent and independent of the choice of subjects or initialization.

These results indicate that the individual subjects' network analysis gives similar results to the combined healthy group network. However, we need to show the effect of sample size on the group network computation. The statistically established technique for approaching these problems is via rigorous bootstrap sampling. Basically, we want to answer the question: how reliable is the average network, and how would our results change if a smaller or different sample was used? These questions can be answered by repeated resampling with replacement, i.e. bootstrap technique. We implemented this technique in MATLAB, once for each iteration of Algorithm A6, and report the mean objective function (blue curve) as well as the 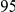
% confidence interval around it (red curve). This is shown in Figure S4, and demonstrates that the objective function for the average network is in fact a very robust and consistent result, which simply could not have arisen due to chance.

As a final result regarding wiring cost minimization, we performed a pairwise t-test to see whether the starting configuration, which is random, is statistically different from the ending configuration. The test returned a p-value of 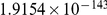 for the wiring cost and 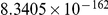 for the similarity measure, indicating that both quantities are highly statistically significant.

**Figure 4:**
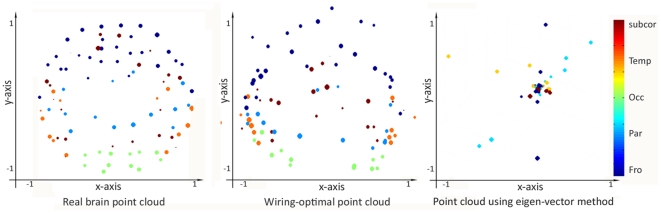
Brain configurations using various algorithms. Point cloud of the brain (left) and the wiring-optimally configuration (middle) are shown, color coded by lobe and sized according to node strength. For comparison the eigen-vector method [11] is also being shown (right).

Figure 4 shows a point cloud of the centroids of cortical and subcortical regions mapped onto the unit sphere for the real brain (left) and the wiring-optimal configuration (right). The points are color coded by lobe and their size denotes node strength. The view is an approximately coronal projection of the brain. Notice how the points tend to locate themselves in roughly the same configurations as the brain, where points belonging to each lobe tend to stay together. The lobes are anatomically correct in relation to each other, although there appear significant variations within the lobes. As a point of comparison, we implemented the exact solution proposed in [11] for this problem in terms of the small eignevectors of the graph Laplacian (see Related Work in Discussion) - this is also presented in the figure. It can be seen that the results of the eigenvector approach are disappointing, and lead to the nodes clustering in s few collinear clusters.

**Figure 5:**
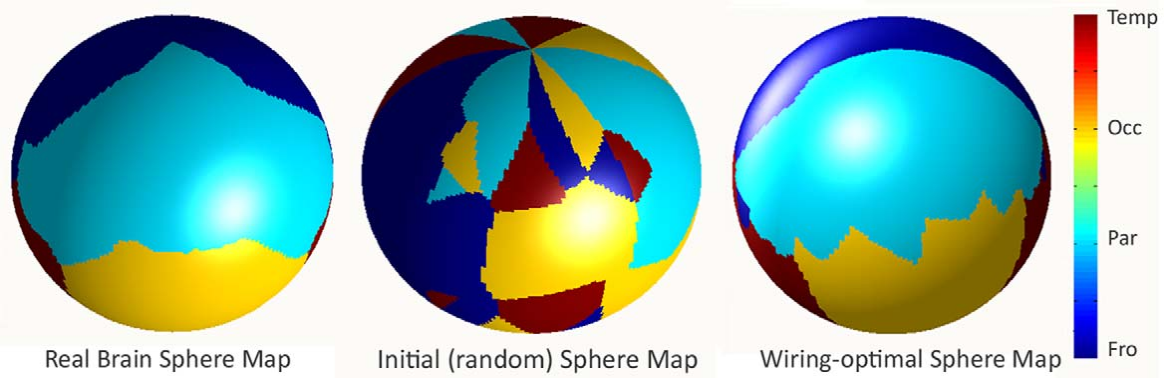
3D rendered unit sphere surface of the brain and the wiring-optimal configuration. This is an approximately coronal view overlooking the parietal lobe, color coded by lobe.

**Figure 6:**
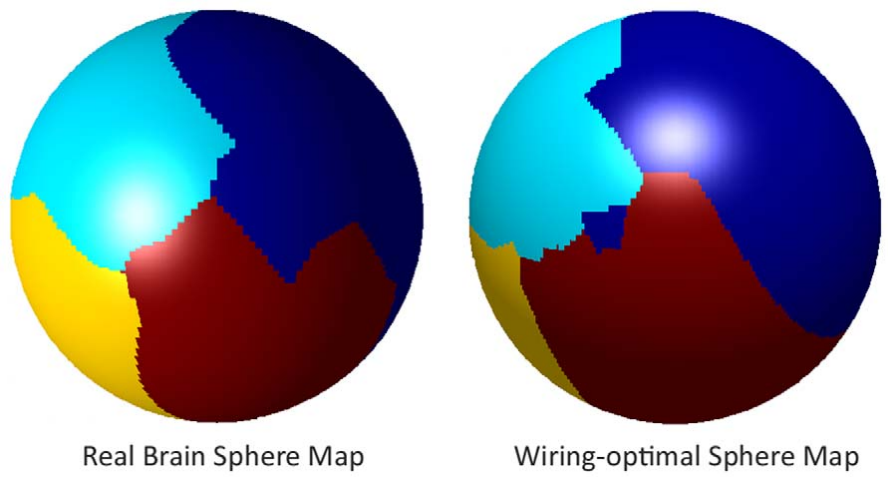
Sagittal view of previous figure. This is an approximately sagittal view showing the intersection of the parietal, temporal and frontal lobes. The dataset being shown is the same as Figure 5.

**Figure 7:**
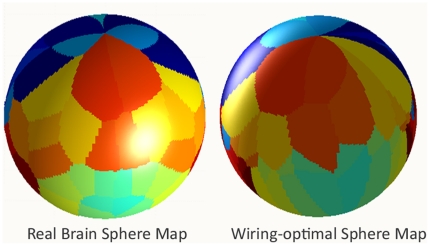
3D surface color coded by cortical region. The coronal view and the dataset is identical to that of Figure 5.

Figure 5 shows 3D rendered surfaces of the unit sphere for the brain as well as for the optimally-placed nodes after running the minimization algorithm described in Methods. The surface is color coded by the lobe to which each point belongs. This view is approximately coronal, showing the parietal lobes in each hemisphere, with both the parieto-frontal (above) and the parieto-occipital (below) interfaces visible. Note the striking similarity in appearance of the two configurations. In figure 6 the same surfaces are shown in a sagittal view. In figure 7 the same surfaces are shown, but this time color coded by each individual cortical regions. The view is approximately the same as above, showing regions in the parietal lobe and adjoining regions in the frontal and occipital lobes. Again note the similarity in appearance. In Figure S2 another example is shown, starting from a different random configuration, but producing remarkably similar optimal surface. We repeated this analysis multiple times, and observed the same tendency each time.

**Figure 8:**
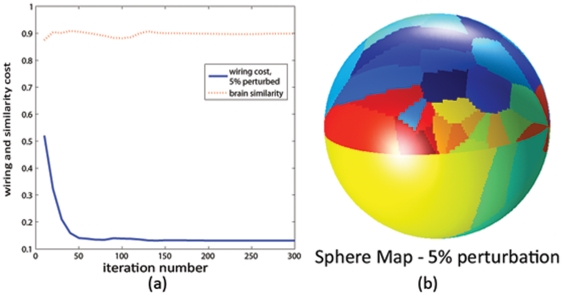
Effect of random perturbations. (a) Minimization of the wiring cost and similarity measure for a randomly perturbed connectivity matrix - 5% rewiring. (b) Corresponding optimal surface map, color coded by cortical region. There is a noticeable mismatch to the brain sphere map.

In order to assess whether changes in connectivity and topology can affect the anatomical placement of regions, Figure 8 shows results for a slightly modified network, where we randomly rewired 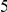
% of brain connections. The resulting “optimal” surface has no resemblance to the brain sphere map, implying that connectivity determined anatomy. In Figure S3 we show another example of the perturbed connectivity matrix with 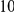
% rewiring.

### Optimal wiring cost of random networks: varying connectivity and placement

**Figure 9:**
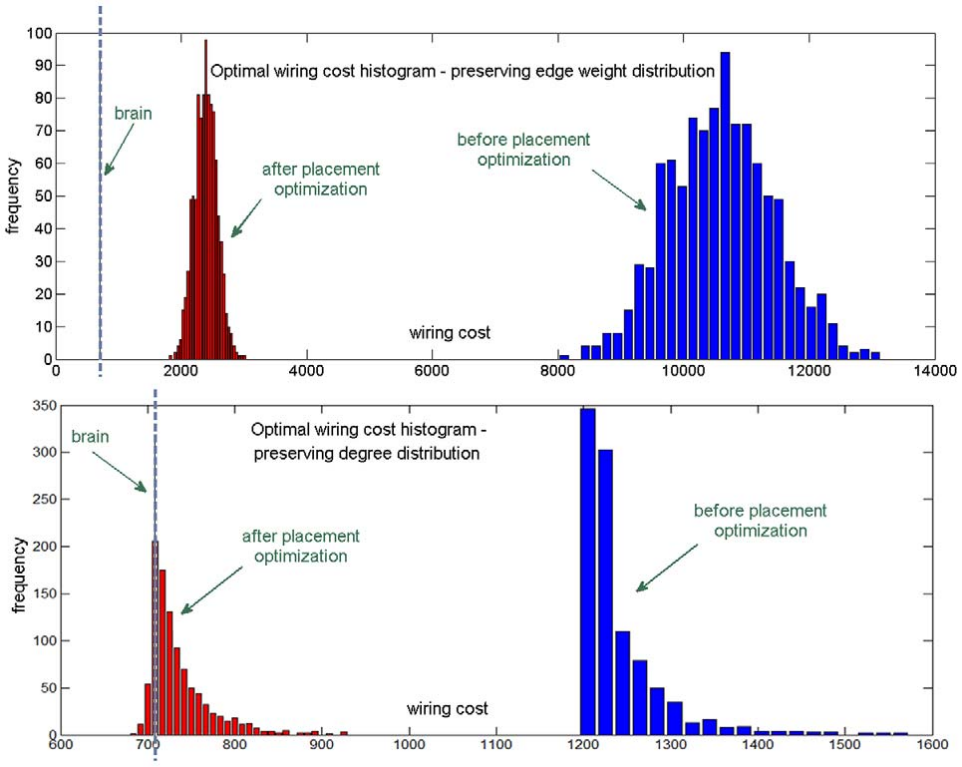
Wiring cost minimization for 1000 random networks. Histogram of wiring cost of random networks at the brain's configuration (blue) and after placement optimization (red) for 1000 random networks, with the same edge weight distribution as the brain (top), and the same weighted degree distribution (bottom). Wiring cost of the real brain network is shown by the dotted vertical bar for comparison. Placement optimization greatly reduces wiring cost for any random network, sometimes giving cheaper-than-brain wiring cost (bottom).

Having determined that the wiring-optimal configuration can be computed using Algorithm A6, we wish to determine if its application on a random network will reduce its wiring cost, and whether such optimal wiring cost is comparable to the brain's. Histograms of starting (brain-like) and optimal configurations are shown in Figure 9 for 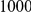 random networks. Placement optimization clearly gives reduced wiring cost of random networks, in some cases (bottom panel) lower than the brain's. Note: wiring cost numbers are different from those in Figure 1, because here the sphere-mapped rather than the real brain configuration is used.

## Discussion

### Brain connectivity network (nearly) minimizes wiring cost

From the first set of random simulations it can be concluded that in comparison to a very large number of random networks equivalent in important ways to the brain network, the latter produces a much smaller wiring cost (Figure 1). This cost is highly statistically significant, as demonstrated by both the bootstrap permutation testing as well as the individual subjects' t-test. It is very difficult to generate an equivalent random network with lower wiring than the brain.

However, it is possible to obtain cheaper networks using specifically designed algorithms (Figure 2). This conclusion was also reached in previous reports [7], [19] (see Related Work). Clearly, wiring optimality is not the only consideration in the brain, which must balance it with favorable topological properties suitable for large-scale parallel processing; these properties are frequently in conflict with wiring economy. Indeed, the cheaper wiring cost of these contrived networks comes at the cost of path length; in fact, many such cheap networks are disconnected.

### Connectivity Determines Anatomy

The second analysis, while confirming again that the brain (nearly) minimizes wiring cost given the network topology and connectivity that it has, is in many ways more revealing. It starts with known brain connectivity and ask the question, what configuration would the nodes take if they were free to roam the putative brain manifold? It is intriguing that we can begin with any randomly placed configuration, but by minimizing the wiring cost (2), somehow we always end up in a configuration that looks remarkably like the brain. The observed node placement in the brain is a direct consequence of wiring cost constraints imposed by the particular choice of network topology/connectivity favored by the brain. Therefore, the wiring economy principle, when applied to the observed brain connectivity, uniquely yields the observed brain anatomy.

However, the rewiring results (Figures 8 and S3) reveal that the wiring economy principle, when applied to observed brain anatomy, is not sufficient to uniquely reproduce the observed connectivity. If the brain had a different topology/connectivity, its anatomy might have been quite different. Figures 8 and S3 suggest that perturbations from the putative brain network lead to non-brain like configurations, and support the claim that it is connectivity that is primarily optimized in the brain, whose lowest-wiring embedding uniquely determines anatomy. This is confirmed by results of Algorithm A4 in Figure 2, which show that cheaper-than-brain network topologies are possible under the brain's anatomic configuration. Clearly, the brain's observed topology is not a unique or even optimal result of wiring minimization, given the brain anatomy. The placement-optimized wiring cost results in Figure 9 also support this claim, showing that the brain's anatomic configuration is almost always sub-optimal for random networks. Further, there exist many random networks (bottom panel) with cheaper-than-brain wiring cost under a non-brain-like configuration. Finally, the work of Van Essen and Stevens [6], which posits that highly connected regions are naturally drawn to each other during development due to axonal tension, also supports our finding that connectivity determines anatomic placement. If the reverse were true, a tension-mediated developmental process could not easily account for it.

If one believes that wiring economy is a central organizing principle of the brain, then from the combined weight of these results it may be plausibly concluded that connectivity determines anatomy, and not vice versa.

### Related Prior Work

Although both the rewiring problem (constant placement, varying connectivity) and the placement problem (constant connectivity, varying placement) have appeared previously, presented work is the first time both these approaches have been evaluated on human data. Although neural-scale component placement has been analyzed [10], [12], we are not aware of studies of human whole brain networks comparable to ours. An exactly solvable quadratic formulation was proposed in [11], under both external and internal constraints. The external constraints impose “hard-wired” costs associated with sensory-motor regions, a concept we find both impractical to impose and difficult to justify in the human brain. Their internal constraints are simply that the nodes lie on the unit sphere, and that their centroid be at the origin. This has a very big advantage, in that the solution is explicitly and elegantly given by the 3 eigenvectors of the Laplacian of the connectivity matrix corresponding to the 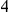
th to 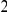
nd smallest eigenvalues - fact first noted by Hall et al. [20]. However, this advantage may be weighed against these disadvantages: a. the centroid-at-origin constraint does not properly capture the that nodes should not occupy the same or close by positions, b. the unit sphere constraint does not allow for subcortical structures, c. the results in [11] are limited and on human brain data (Figure 4(c)) are disappointing. Since the method cannot handle subcortical nodes, the latter were removed from the network during the eigenvector calculation.

Component placement in Macaque and *C. elegans* brains was analyzed using tracer data [7]. There are several important differences in methodology and data compared to our work. First, [7] does not account for weighted connection strength, treating the metabolic cost of a weakly-connected pathway the same as a strongly connected one except that they could assume one of 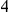 possible ordinal (rank) values from 0 to 3. Since this data does not admit real-valued connectivity weights, it could lead to sub-optimal component placement. Second, our methodology involves an efficient descent-based deterministic minimization algorithm, compared to the stochastic simulated annealing method used in [7]. Finally, our methodology allows network nodes much more freedom to explore alternative configurations, since a node can be located anywhere on the manifold. In contrast, [7] fix node locations, and only allow them to exchange places, a triad at a time. A similar location rearrangement study was reported by Klyachko et al [21] on Macaque brain, although of more limited scope than [7]. They report that by randomly permuting 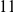 nodes within the macaque brain they could not find a cheaper wiring configuration than the real configuration. The study, however, was limited by the small number of nodes considered and the 4-level ordinal connection weights.

A study of dMRI-derived human brain connectivity, similar to our rewiring experiments, by Bassett el al. [19], found that human and nematode brains share common fractal scaling properties with VLSI systems, a result which may be explained by wiring economy. Like [15] and [7] however, their wiring cost does not truly admit connection strength, which were binary valued. We believe that the topology and statistics of unweighted networks are very sensitive to the thresholding step, rendering any conclusions controvertible. In this paper we explicitly incorporate connection weights in our wiring cost measure. By avoiding the binarization process our results might be more robust and generally applicable. Even so, our results generally support their main conclusion that although the brain is efficiently wired, it does not strictly minimize wiring cost. Figure 2 is similar to the wiring result in [19], but we show, additionally, that the cause of cheaper wiring is that inter-hemispheric connections are rerouted to subcortical connections, leading to extremely large path lengths which make such networks inviable as computing devices.

### Limitations

Wiring cost (1) assumes that the connection weight of an edge is a true measure of the number and density of fibers that connect it, ignoring the possibility that some pathways, due to aggregation of fibers into coherent bundles (e.g. corpus callosum), might in fact represent less metabolic cost that other, more dispersed fibers. The true fiber distance between any two nodes is not necessarily Euclidean. Although we have access to tract-based distance of all connections from tractography, we decided not to use it for two reasons. First, such a measure is clearly not applicable for the placement study which involves arbitrarily moving the nodes on the manifold. Second, we wish to avoid biasing the model by prior knowledge of tract geometry, and show that even in absence of detailed knowledge of white matter connectivity the brain topology is near-wiring-optimal. The aim here is to show that geometric embedding of nodes on a simple manifold can roughly reproduce brain anatomy.

Our results rely on mapping the convoluted brain surface to a sphere. We believe this simplification is preferable to constraining nodes to lie on the actual brain manifold, which might simply bias the algorithm in favor of producing brain-like configurations. Further, we do not believe totally unconstrained models to be appropriate because there exits metabolic constraints due to which closed-sheet configurations are preferred over arbitrary 3D configurations [12]. Brains have other evolutionarily-determined goals whose implementation on biological substrates has physical and biological constraints [22], [23]. Since these external constraints are impractical to enforce explicitly, we have substitute them with the manifold constraint. Even with the manifold constraint, the nodes still have a tremendous amount of freedom in choosing their geographical location. The fact that they end up in a brain-like fashion is still quite remarkable. A related question is the choice of a smaller sphere for subcortical structures. It is well known that the subcortical grey matter in the brain does not lie on the cortical ribbon, and in fact comes from a much older stage of brain evolution. We found that the best way to depict this situation would be to allow subcortical nodes to rest on a smaller sphere. The choice of half diameter is somewhat aribtary, but we found that this choice is not terribly important. The results are largely unchanged even if a different diameter is used.

#### Surface rendering

All out visualizations were done on the unit sphere. Obviously a more involved visualization scheme, whereby wiring-optimized configuration is mapped back onto the actual brain manifold, might produce more realistic looking images. However, we believe this will simply aid visualization, without producing additional insight or better results.

There are several methodological limitations. First, network extraction requires a lengthy, involved process [24], whose deficiencies are inherited by our results. There is a well-known distance bias in current tractography algorithms such that the probability of tracking a connection decreases with increasing length of the tract. We do not believe this affects the conclusion that the brain has near-optimal wiring, because of similar results in unweighted networks [15], [19], where the distance bias is weaker due to the thresholding step. Many biological properties of the cortex can falsely increase or decrease the measured number of tracts in many cortical regions. For example, areas in which fibers cross may have fewer reconstructed tracts than areas where no fibers cross simply due to the inadequate tractography and not due to the underlying biology. Similarly, tracts close to the corpus callosum are often tracted into the corpus callosum when they are known from histological studies not to pass through the corpus callosum. In light of these limitations, the underlying assumption that tractography-derived connection weight is a true measure of the number and cross section area of fiber pathways cannot be said to be completely true. The entire field of tractography is an active and evolving area of research, and we feel many of these deficiencies will resolve themselves over time. Already, the issue of crossing fibers is ameliorated by our use of a large number of diffusion directions and advanced q-ball reconstruction. However, we note that ultimately this paper is not about the network extraction methodology, but rather its application to address wiring cost. We think our results are strong enough to survive any reasonable future consensus on how connection weights should be computed.

With only 90 nodes, our network might be considered “lumpy”; it is unclear if a finer-scale network with more nodes will yield improvements. Finally, variations in size and shape of cortical regions is not accounted for in our model. For this reason we would prefer that the ROI size be equal, but unfortunately atlases faithful to clasical brain anatomy do not tend to have this property. Although ROI size may be incorporated within Eq (2), but decided against it for 2 reasons: a. the size information is quite noisy and unreliable, b. it introduces an unnecessary complication to our formulation, which is characterized by its uncommon simplicity (only using wiring cost and electrostatic force). Nevertheless, the size variation issue may not be as important as it might appear, because cortical (large ROIs)and subcortical (small ROIs) nodes are already treated differently in our formulation.

## Methods

### Model

The brain network is already embedded in Euclidean space because each node represents an anatomically pre-determined cortical or subcortical region. We assign each node the location of the centroid of the region it is supposed to represent; this information is obtained directly from the atlas. Let us define the pairwise distance between any two nodes as 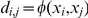, where 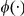 is some distance metric, and 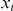 is a 3-vector of spatial coordinates 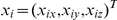. In this paper we use the usual Euclidean distance metric 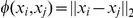. Given a distance metric 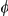 and connectivity 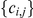 for 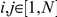 of a 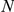-node network, the wiring cost is specified by
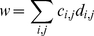
(1)This definition of wiring cost is natural under the assumption that the metabolic cost of a brain network is proportional to the total volume of the fibers that connect its nodes. Intuitively, since available brain volume is limited by the human anatomy, there must be a cost for admitting any new volume of connecting fiber. Several studies [5], [6], [8]–[12] have shown that there are metabolic costs associated with creating and maintaining neuronal pathways. Most, but not all, of these studies report cost proportional to volume. The total volume, in turn, is proportional to the total length of the fibers, weighted by the connection weights of individual fiber pathways. Recently, Chklowskii et al [10] have proposed a non-linear dependence on fiber length using mathematical modeling. However, this does not appear to be thoroughly validated at this point. We also assume that the connection weight of an edge is a true measure of the number and cross section area of fibers that connect it. For a discussion on this and other possible limitations see Discussion.

Let us now consider the cheapest geometric embedding of the observed brain network. Since human brains have highly convoluted shapes due to the pressure to fit a large cortical sheet within a bound cranial volume, it is reasonable to map the cortex to a spherical shell to which it is topologically equivalent. By performing operations on this shell we keep most of the essential features of brain anatomy while discarding confounding influences like cranial pressure and volume, blood flow, etc. Therefore we declare the surface of a unit sphere as the manifold on which all cortical nodes are allowed to move. Similarly, we constrain subcortical and deep brain structures to the surface of another sphere, this one half the diameter of the cortical sphere. This smaller radius shell is somewhat arbitrary, but its choice did not appear to strongly affect our results. The final constraint is to force the nodes to belong to the left and right hemispheres based on prior anatomic information. No further constraints were placed on node placement.

A suitable cost function must incorporate not only wiring cost but also ensure the nodes are placed at sufficient distance from each other, since they have non-zero volume. Without this constraint all node locations will coincide for minimum wiring, which is anatomically impossible. This was achieved by adding a very simple electrostatic repulsion term between nodes following the canonical 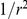 law, forcing them to prefer being located as far away from each other as possible on the manifold. The balance between wiring cost and electrostatic repulsion gives the objective function
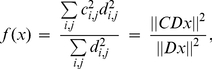
(2)where we have collected all location vectors 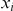 in the large vector 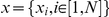. The right-most expression indicates that the objective function is a ratio of a weighted and an unweighted 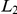 norm of the set of node locations 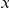, for an appropriately defined pairwise-distance operator 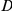. Note that this formulation is a ratio, and therefore scaling-free: it does not depend on the relative strength of the contribution of the numerator and denominator. Our task is to minimize this objective function under the above manifold constraints and find the wiring-optimal configuration:
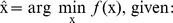
(3)

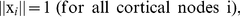
(4)

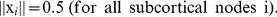
(5)


We now give an efficient minimization algorithm using gradient information to solve (3).

**Theorem 1**: (a) The gradient of 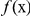 is given by
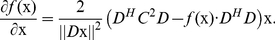
(6)


(b) Let us combine the left hand sides of both constraints (4) and (5) in the *constraint vector* 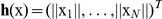. The gradient of 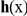 is given by
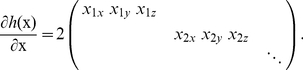
(7)


*Proof*: (a) By derivative of ratio rule,
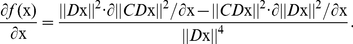
(8)


Now 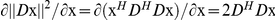, and 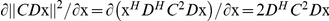. Proof follows after simplification. (b) For the second part, 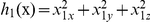, giving 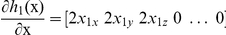. Similar expressions exist for all 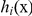, which completes the proof.

Since the gradients of both the objective function (2) and the constraint vector 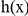 specifying the nonlinear constraints (4) and (5) are explicitly known, this information can be used to speed up the minimization routine. We implemented an active set constrained minimization algorithm [25], which relies on estimates of the gradient vectors of the objective function as well as the constraints. Although these gradients can be computed numerically using finite differences, the cost of doing so becomes prohibitive due to a large number of function calls for evaluating 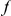. The problem is made even more challenging due to the highly multidimensional search space and the nonconvex nature of the objective function, requiring a large number of iterations for convergence. Instead we supply the gradient information explicitly using Theorem 1, which enables fast and efficient minimization. Note that due to non-convexity of (2), a global minimum is not guaranteed to be found. However, a strong local minimum can be found. Results of the minimization (see Results) show almost total independence from the initial guess after 200 iterations, suggesting that in fact the global minimum might have been found. We have therefore concluded that the minimization landscape is relatively well-behaved, and the addition of explicit gradient information has allowed robust convergence insensitive to initial guess.

### Subjects and MR imaging


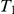
-weighted structural MR and High Angular Resolution Diffusion Imaging (HARDI) data were collected on 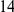 healthy adults with an average age of 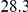 years (range 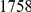 years; standard deviation 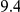 years) on a 3 Tesla GE Signa EXCITE scanner (GE Healthcare, Waukesha, WI, USA) as part of an existing ongoing study at our institution, and approved by our Institutional Review Board. Written informed consent was obtained in accordance with guidelines set forth by the Declaration of Helsinki. HARDI data were acquired using 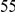 isotropically distributed diffusion-encoding directions at 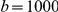 s/mm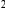 and one at 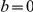 s/mm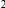, acquired at 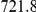-mm thick interleaved slices with no gap between slices and 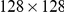 matrix size that was zero-filled during reconstruction to 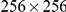 with a field of view (FOV) of 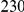 mm. The structural scan was an axial 3D inversion recovery fast spoiled gradient recalled echo (FSPGR) T1 weighted images (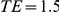 ms, 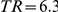 ms, 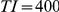 ms, flip angle of 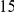
°) with 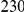 mm FOV and 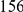 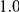-mm contiguous partitions.

### Network extraction

Diffusion tractography processing closely followed [24], and is briefly summarized here. The structural and diffusion MR volumes were co-registered using the Individual Brain Atlases using Statistical Parametric Mapping (IBASPM) [26] and Statistical Parametric Mapping (SPM5) [27] software packages in MATLAB. The structural volumes were then parcellated into cortical structures and the brain atlas was created using Automatic Anatomical Labeling (AAL) software [28]. The resulting 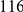 parcellated cortical structures were used to seed corresponding regions in the diffusion volume, and probabilistic tractography was performed. The strength of connectivity between any two gray matter structures is basically the cross section of the fibers going between them.

Our analysis considers only the cerebrum, therefore the 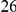 cerebellar structures and their connections are removed, giving a symmetric 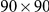 connectivity matrix 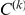 for the *k*-th subject with 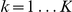, whose entries are individual connection strength 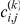 between any two nodes 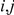. We deduce the *joint* connectivity matrix 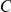 for all 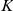 subjects, and use the joint matrix for further analysis. In addition, we repeat our analyses on individual subjects and perform permutation tests to obtain statistical significance values of the wiring cost analysis of the joint matrix.

Prior studies threshold the real-valued connectivity and convert them to unweighted links [15], [17], [19]. Since resulting topology is sensitive to the threshold, these studies have advocated reporting network measures over a large range of thresholds. Unfortunately, the threshold range is quite arbitrary; this approach is neither statistically optimal nor does it ensure that viable connections are not obliterated by overly high thresholds. Here we employ a principled method based on hypothesis testing following [29], [30], to decide whether a connection is statistically viable:

1. Calculate joint variance 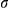 of all non-zero entries in the upper triangular part of the 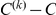 matrices.
2. Perform hypothesis testing on sample consisting of entries 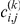, 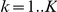, with zero mean, variance 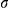 and some significance value 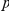. This determines if the hypothesis that distribution for 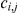 is centered at zero can be refuted. If it can, we keep all the 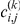, and if it cannot, we set them to zero in the modified matrices 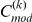.
3. Repeat steps 1 and 2 with matrices 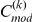. This is done until average matrix 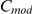 does not change anymore.

In this paper we use the average connectivity obtained from 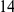 healthy subjects after significance testing at the level of 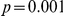.

### Wiring cost I: Same node placement, varying connectivity

First the wiring cost of the the brain network according to Eq. (1) was determined. Then a large number of random networks were generated and their wiring cost was computed; histograms of both were computed. For the purpose of this study the random networks are constructed in three ways:

#### Algorithm A1: Preserving edge weight distribution

Each random network was drawn such that its edge weights have the same histogram as the extracted brain network. This was done by redistributing the edge weights of the brain network randomly within the upper triangular portion of a connectivity matrix. The lower triangular portion was simply the transpose of the upper triangular portion, resulting in a symmetric matrix, hence an undirected graph. Random networks so obtained have the same number and weight of edges as the brain network; the difference is that the network topology is allowed to be arbitrary, giving rise to networks whose degree distribution and other network properties might be quite different.

#### Algorithm A2: Preserving weighted node degree distribution

Each random network was drawn in such a way that it not only maintained the same edge weight distribution as the brain network, but also maintained the same (weighted) degree distribution. This was achieved using a matrix randomization *MaskMetropolis* [31] that preserves row and column sums; networks with the same weighted degree distribution for each node have connectivity matrices with approximately the same row and column sums. Starting from the brain network, we iteratively perform *MaskMetropolis* operations to generate random networks with the same row and column sums, taking care to preserve symmetry. Random networks generated with this method are relevant because they preserve the weighted degree of each node; the weighted degree of each node roughly corresponds with its anatomical size [30]. Therefore it is reasonable to constrain node sizes so they don't vary far from what is physically possible.

#### Algorithm A3: Preserving topology

Each random network has the same edge weight distribution and the same topology as the brain network, but the weights were randomly permuted within the given topology. Each random network has the same edges as the brain, with different edge weights. This case is interesting because it highlights whether the edge weights assigned in the brain are particularly optimal; with the topology held constant, only the edge weights can alter the wiring cost.

It turns out that although cheaper wiring than brain does not arise in above random networks, it is not hard to construct them - only difficult to get them from random sampling. We implemented two specific algorithms to construct low-wiring-cost networks:

#### Algorithm A4: Cheaper than brain networks

Starting from the brain network, randomly permute any pair of edges and keep the new network if its wiring cost is smaller. Repeat this until no new permutation reduces wiring cost. This procedure keeps the number of edges and their weights the same as those found in the brain.

#### Algorithm A5: Cheapest possible network

This algorithm is a simple extension of one first proposed in [7], which applied only to unweighted networks. It constructs the cheapest possible network keeping the same edge weights as the brain but without preserving any other network property. First, create a minimum spanning tree so all nodes are connected. Then separately sort pairwise distance between nodes and edge weights between nodes. Begin assigning the largest wedge weight to the node pair with the smallest distance. Keep adding edges in this way until all edges are assigned.

### Wiring cost II: Same connectivity, varying placement

The following algorithm finds the node placement which minimizes (2):

#### Algorithm A6

Start with a random placement of nodes on the 2-shell manifold. At each iteration, compute the gradient of the objective function as well as the constraint function (Theorem 1). We implemented an active set constrained minimization algorithm [25] using prewritten scripts in MATLAB, version 7.8.0 (Mathworks Inc, Natick, MA) on a desktop computer with 2.6 GHz processor and 4 GB RAM. The algorithm was allowed to run for a maximum of 300 iterations or convergence to within a tolerance of 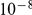, whichever occurred earlier.

The minimum-wiring node configuration was plotted on the unit sphere as follows. A point cloud residing on the unit sphere was generated in spherical coordinates. Each point in the cloud was assigned the label of the node closest to it in terms of angular distance, i.e. the Riemannian distance on the unit sphere. For the purpose of visualization subcortical nodes were not shown. The 3D surface of both the brain and the placement-optimal configuration were rendered using the *surf()* tool in MATLAB. Regions were color-coded by lobe and by region from the atlas.

Finally, we wish to determine if the wiring-optimal configuration of a random network will reduce its wiring cost, and whether such optimal wiring cost is comparable to the brain's. This is done by combining algorithms A1 and A6, as follows:

#### Algorithm A1+A6 and A2+A6

Random networks were drawn according to Algorithms A1 and A2. Their wiring cost under the starting configuration corresponding to the sphere-mapped brain anatomy was computed. Then Algorithm A6 was applied in order to compute the wiring-optimal configuration. These steps are repeated for 1000 different random networks.

## Supporting Information

Figure S1 Significance thresholded connectivity matrix for p = 0.001.
(1.02 MB TIF)

Figure S2 Another example of sphere surface of the brain and the wiring-optimally configuration, color coded by cortical regions.
(0.32 MB TIF)

Figure S3 Another example of randomly perturbed connectivity matrix - 10% rewiring, color coded by lobe. Notice complete lack of resemblance to brain sphere map.
(0.38 MB TIF)

Figure S4 Bootstrap for testing significance of the objective function under random sub-sampling of subject data. The mean of the objective (blue curve) is shown at each iteration, as well the 95% confidence interval. In order to visualize the different curves only a zoomed-in section of the plot is being shown. Note the extremely small range, implying that the wiring cost result is consistent, robust, reproducible, and shows little sampling effect.
(0.69 MB TIF)

Supporting Information S1 Wiring optimization for six individual subject's connectivity matrices. Although there is significant noise and variability in connectivity of individual subjects, every single subject configuration is largely comparable to and consistent with the combined group average result.
(4.83 MB TIF)

Supporting Information S2 Binary MATLAB file connectivity-matrices.mat, which includes all matrices for 14 healthy subjects after significance thresholding.
(0.04 MB ZIP)
