# Supplementary figures and images for: The Wiring Economy Principle: Connectivity Determines Anatomy in the Human Brain
Source: PLoS One. 2011 Sep 7;6(9):e14832. doi: 10.1371/journal.pone.0014832 (PMC3168442; doi:10.1371/journal.pone.0014832)

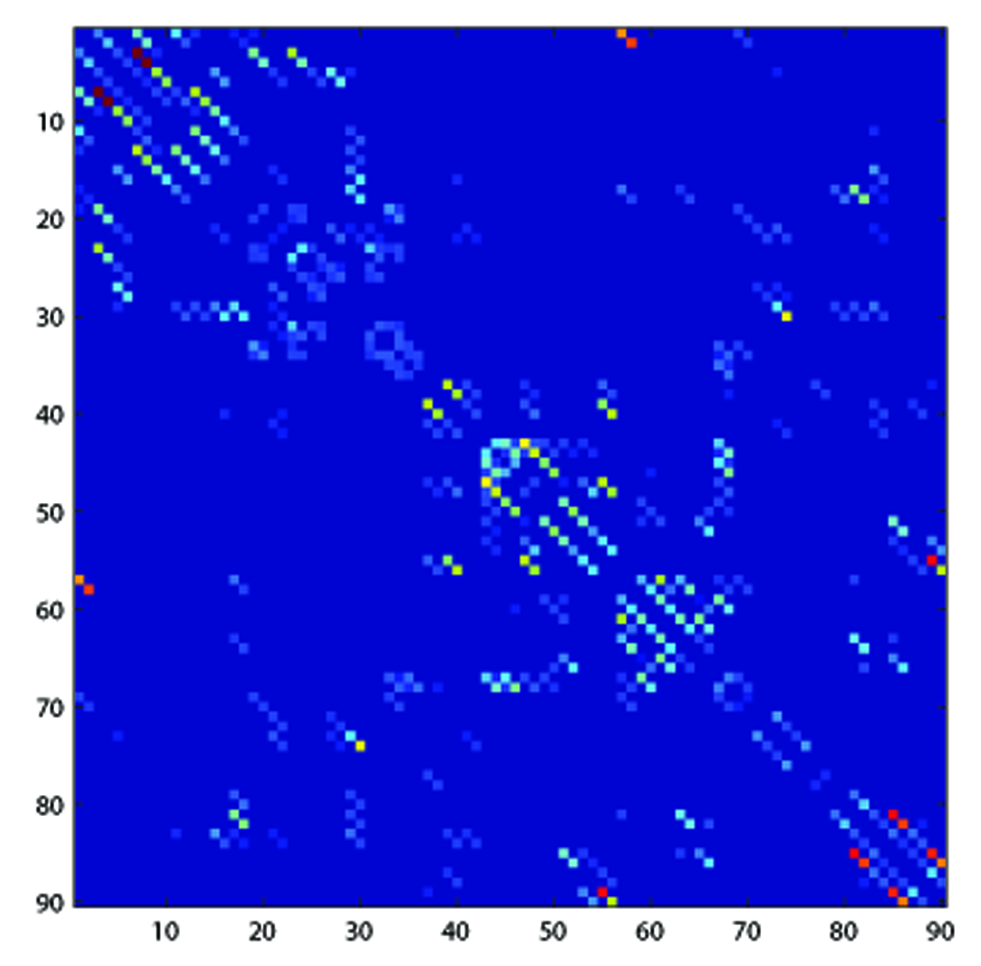

Supplement: Figure S1 — Significance thresholded connectivity matrix for p = 0.001. (1.02 MB TIF) [file pone.0014832.s001.tif]

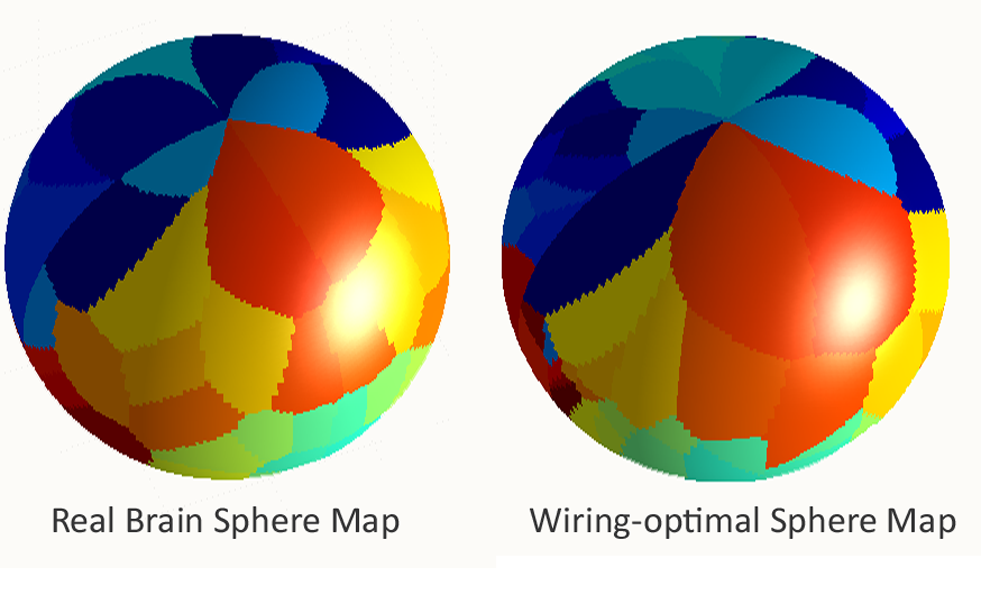

Supplement: Figure S2 — Another example of sphere surface of the brain and the wiring-optimally configuration, color coded by cortical regions. (0.32 MB TIF) [file pone.0014832.s002.tif]

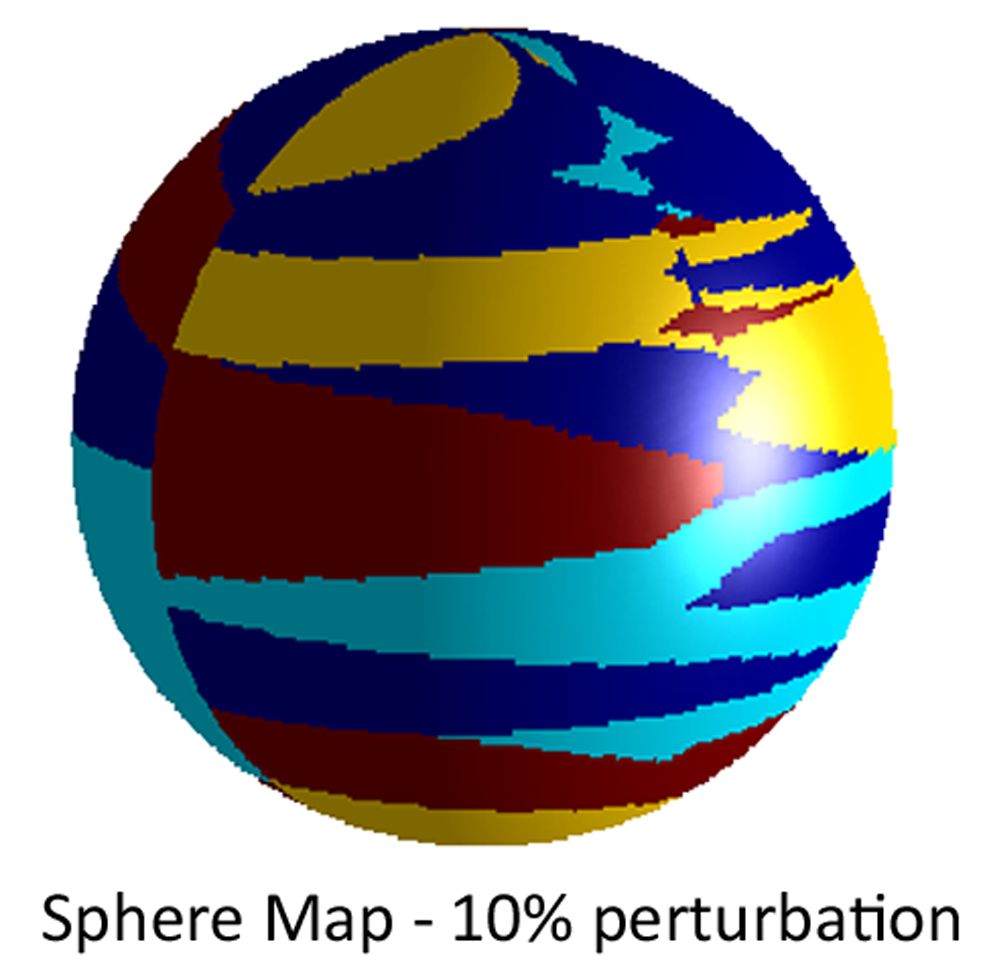

Supplement: Figure S3 — Another example of randomly perturbed connectivity matrix - 10% rewiring, color coded by lobe. Notice complete lack of resemblance to brain sphere map. (0.38 MB TIF) [file pone.0014832.s003.tif]

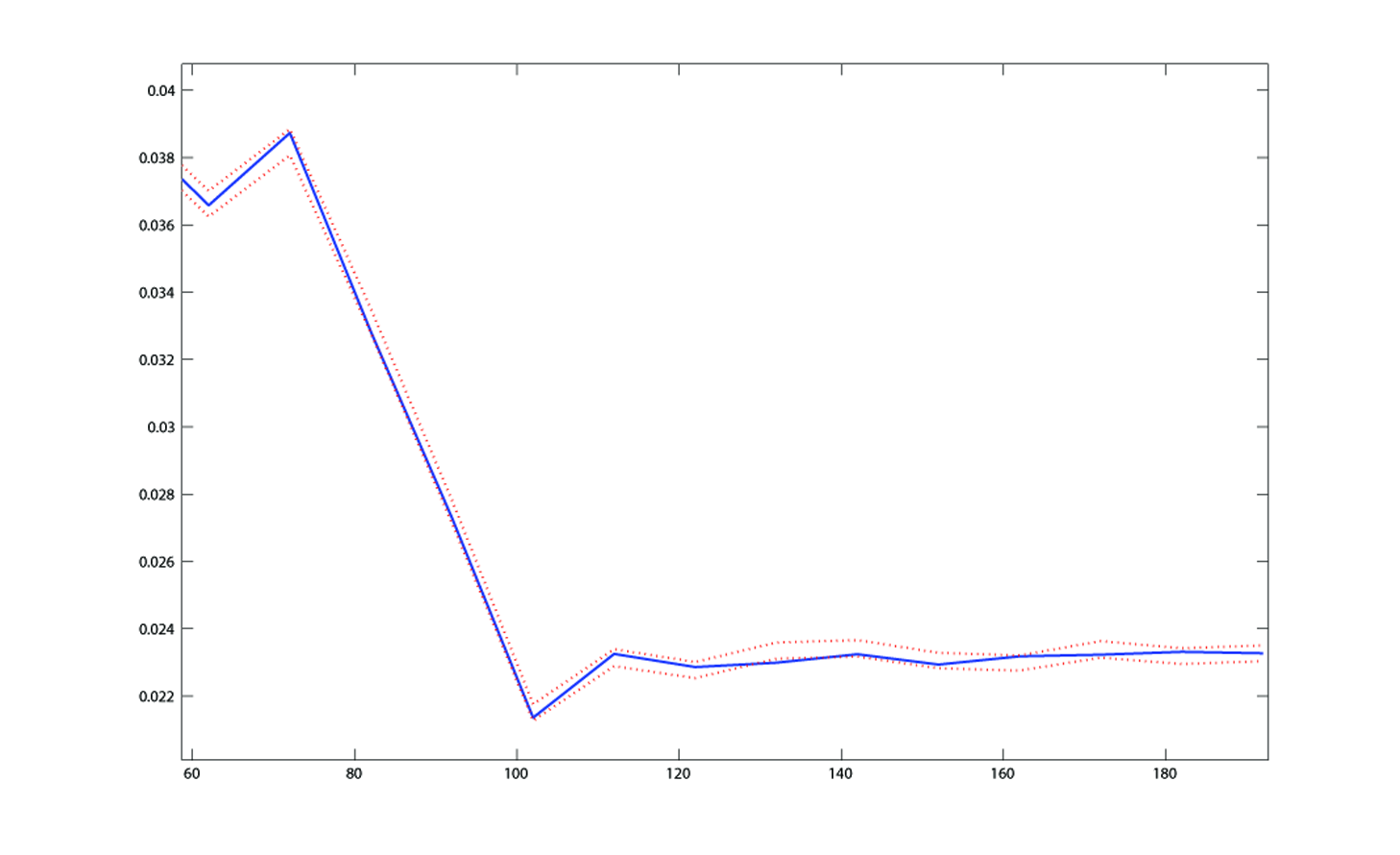

Supplement: Figure S4 — Bootstrap for testing significance of the objective function under random sub-sampling of subject data. The mean of the objective (blue curve) is shown at each iteration, as well the 95% confidence interval. In order to visualize the different curves only a zoomed-in section of the plot is being shown. Note the extremely small range, implying that the wiring cost result is consistent, robust, reproducible, and shows little sampling effect. (0.69 MB TIF) [file pone.0014832.s004.tif]

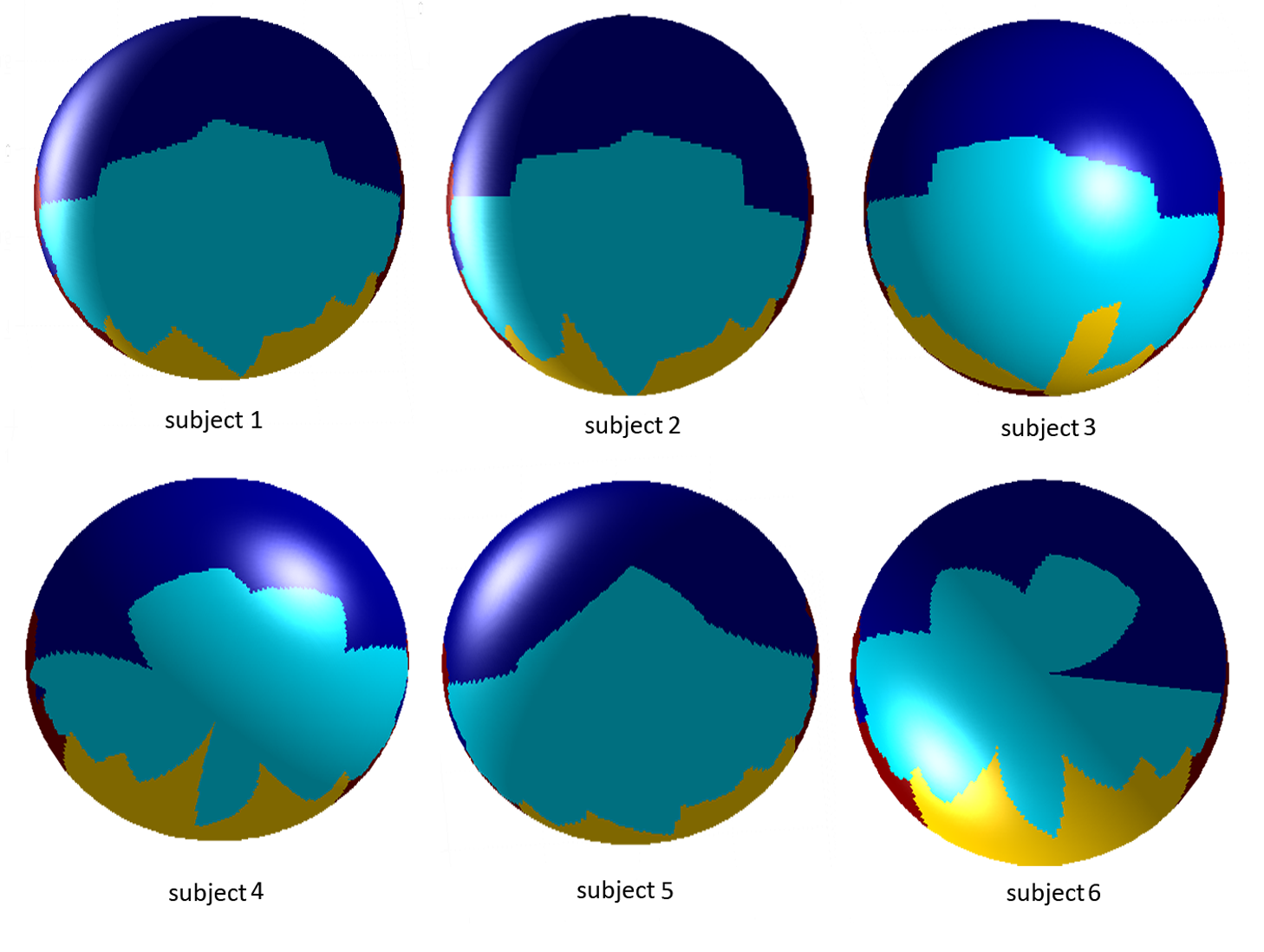

Supplement: Supporting Information S1 — Wiring optimization for six individual subject's connectivity matrices. Although there is significant noise and variability in connectivity of individual subjects, every single subject configuration is largely comparable to and consistent with the combined group average result. (4.83 MB TIF) [file pone.0014832.s005.tif]
